# The zinc transporter ZIP9 (Slc39a9) regulates zinc dynamics essential to egg activation in zebrafish

**DOI:** 10.1038/s41598-020-72515-4

**Published:** 2020-09-24

**Authors:** Aubrey Converse, Peter Thomas

**Affiliations:** Marine Science Institute, The University of Texas at Austin, 750 Channelview Dr., Port Aransas, TX 78373 USA

**Keywords:** Reproductive biology, Metals, Genetic engineering

## Abstract

The zinc transporter ZIP9 (SLC39A9) was recently characterized as a membrane androgen receptor in various teleost and mammalian cell models. ZIP9 shows the highest expression in ovaries of teleosts, a tissue in which both androgen signaling and zinc dynamics have significant roles. To examine the role of ZIP9 in ovarian physiology, we generated a ZIP9-mutant zebrafish strain using a CRISPR/Cas9 system. *zip9*^*-/-*^ females showed significant reductions in fecundity, embryo viability, and growth of their offspring compared to wildtype (WT) fish. Furthermore, a high proportion of *zip9*^*-/-*^ eggs failed to undergo normal chorion elevation during activation. In WT eggs, zinc was detected in cortically-localized vesicles which underwent exocytosis upon activation. *zip9*^*-/-*^ eggs showed abnormal cortical vesicle development and had a significantly depressed activation-induced zinc release compared to WT eggs. Moreover, pharmacologically sustained elevation of zinc in WT eggs prior to activation resulted in abnormal chorion elevation similar to that observed in *zip9*^*-/-*^ eggs. These results indicate that ZIP9 is essential for proper zinc modulation during zebrafish egg activation and presents the first evidence of zinc modulation during egg activation in a non-mammalian species.

## Introduction

Recently, the zinc transporter SLC39A9 (ZIP9) was found to possess membrane androgen receptor (mAR) activity^1,2^, and is the only zinc transporter known to have hormone receptor activity. ZIP9 was first characterized as a mAR in teleost (Atlantic croaker) ovarian tissue and has since been shown to mediate nonclassical androgen actions in a number of fish and mammalian cell models. To date, ZIP9 has been shown to mediate androgen-induced apoptosis and survival of teleost granulosa/theca (G/T) cells^1,3^, an apoptotic response in prostate and breast cancer cells^2,4^, migration of prostate and bladder cancer cells^5,6^, and tight junction formation in murine Sertoli cells^7^. In many of these models, androgen activation of ZIP9 results in elevation of intracellular zinc levels which in turn modulates the downstream physiological response^1–4^. However, ZIP9 also mediates several effects in the absence of androgen stimulation including migration of glioblastoma cells^8^, induction of fibrosis in irradiated skin^9^, and B lymphocyte receptor pathway signaling^10^. These studies underscore ZIP9’s potential to mediate nonclassical androgen actions as well as zinc signaling in various tissues to elicit diverse physiological responses.

ZIP9 is primarily expressed in gonadal and brain tissues in Atlantic croaker^1^, which indicates a potential role of this protein in teleost reproductive physiology. While both androgens and zinc play vital roles in ovarian physiology, the function of ZIP9 in mediating nonclassical androgen actions and zinc transport within the ovary remains unclear. We recently reported that ZIP9 mediates androgen-induced pro- and anti-apoptotic responses in croaker G/T cells by coupling to different G proteins^3^. These opposite survival responses are follicle-stage dependent in that G/T cells from early-stage follicles exhibit the anti-apoptotic response while G/T cells from later stage follicles (late vitellogenic) exhibit the apoptotic response. These findings support roles for ZIP9 in mediating survival and growth during folliculogenesis as well as apoptosis during the breakdown of atretic or postovulatory follicles.

Zinc signaling has also recently been shown to play critical roles in vertebrate oogenesis and egg activation. In mammals, zinc mediates events throughout meiotic maturation^11^. In periovulatory murine oocytes, zinc maintains prophase I arrest while zinc chelation or dietary zinc deficiency induces resumption of meiosis but with abnormal spindle configurations and subsequent inability to reach metaphase II^12^. Furthermore, zinc is the most prominent transitional metal detected in late-stage murine oocytes and is actively accumulated during meiosis I resumption^13^. Post-ovulation, a zinc “spark,” or rapid release of zinc to the extracellular space has been shown to accompany the fertilization-induced wave of intracellular calcium in mammalian eggs^14–16^. In zebrafish, the ovary expresses the widest variety of zinc transporters compared to non-reproductive tissues^17^. Zebrafish oocytes also acquire zinc content throughout oogenesis^18^, and zinc salts have been shown to promote germinal vesicle breakdown (GVBD) of zebrafish oocytes^19^. While these findings suggest a role for zinc modulation in zebrafish oogenesis and meiosis, its function remains unclear.

ZIP9’s high expression and the critical roles of both androgens and zinc in the ovary emphasize the need for further examination of ZIP9’s function in ovarian physiology. To address this, we generated the first animal knockout model of ZIP9 using zebrafish. *zip9*^*-/-*^ females had severe reductions in reproductive success compared to *zip9*^+*/*+^ (wildtype) sibling controls which was associated with a high proportion of *zip9*^*-/-*^ eggs that failed to undergo chorion elevation when activated. Since zinc has been found to be important in mammalian species during activation, our initial characterization of this *zip9*^*-/-*^ zebrafish model focused on alterations in zinc dynamics rather than modulation of the membrane androgen receptor functions of ZIP9. We observed that in wildtype meiosis II-arrested eggs, zinc is stored in cortically-localized vesicles that undergo exocytosis upon egg activation. *zip9*^*-/-*^ eggs showed a reduced zinc exocytosis response compared to wildtype eggs, which corresponded with abnormal cortical vesicle morphology. This work provides the first evidence that zinc modulation occurs during egg activation in a non-mammalian vertebrate model and demonstrates that ZIP9 plays a vital role in the zinc regulatory events that allow for proper egg activation in zebrafish.

## Results

### ZIP9 expression in zebrafish oocytes and follicle cells

**Figure 1:**
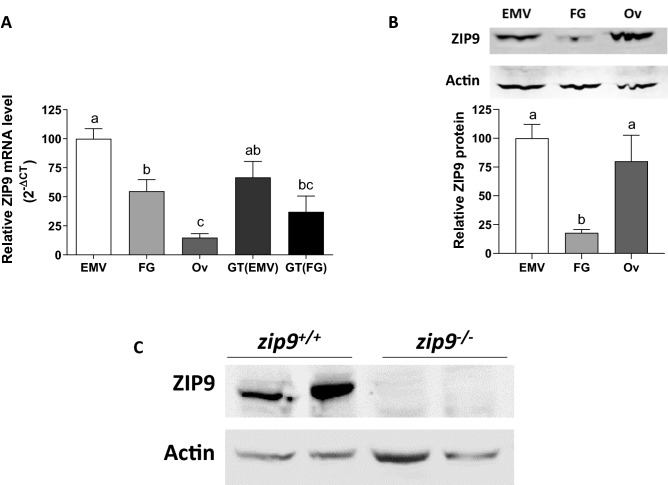
ZIP9 expression in the zebrafish ovary. **(A)** mRNA expression of ZIP9 in different stage wildtype oocytes and follicle cells (n = 4–6). **(B, C)** Western blot analysis of ZIP9 protein expression on the plasma membrane of different stage wildtype oocytes/eggs **(B) **and the plasma membrane fraction of wildtype and *zip9*^*-/-*^ fish ovaries **(C)** (n = 3–4). All data represents means ± SEM. Significance was determined by one-way ANOVA with Bonferroni multiple comparison post-test. Different letters indicate significant differences between treatment groups in the post hoc test (P < 0.05). *EMV* early-mid vitellogenic oocytes, *FG* full grown oocytes, *Ov* ovulated eggs, *GT* (*EMV*) granulosa/theca cells from early-mid vitellogenic follicles, *GT* (*FG*) granulosa/theca cells from full grown follicles.

*zip9* mRNA expression was detected in denuded wildtype (WT) zebrafish vitellogenic oocytes, ovulated eggs, and in ovarian follicle cells (Fig. 1A). *zip9* expression was highest in early-mid vitellogenic oocytes and significantly lower in full grown oocytes (55% expression of early-mid vitellogenic, p = 0.0089) and ovulated eggs (15% that of early-mid vitellogenic, p = 0.0003). *zip9* mRNA expression in G/T cells was not significantly different from that of oocytes from the same follicular stage. ZIP9 membrane protein expression was found to be similar in early-mid vitellogenic oocytes and ovulated eggs but was significantly lower in full grown oocytes (Fig. 1B; Supplementary Fig. 2A,B). This indicates that ZIP9 is developmentally regulated prior to and through the resumption of meiosis.

**Figure 2:**
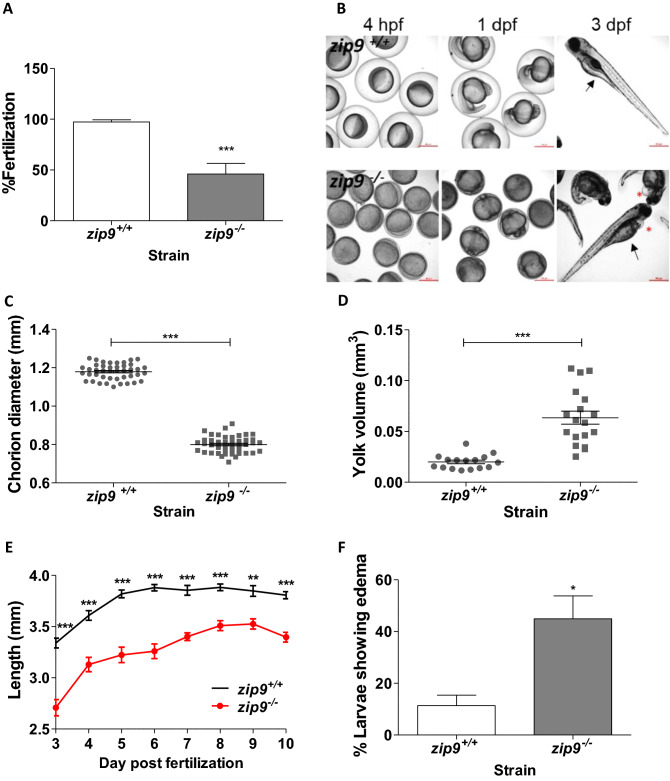
Characterization of *zip9*-mutant phenotype. **(A)** Percent of viable eggs ovulated by *zip9*^*-/-*^ and *zip9*^+*/*+^ females 2 h post fertilization (hpf); n = 13–14 clutches. **(B)** Representative image of *zip9*^+*/*+^ and *zip9*^*-/-*^ embryos at 4 hpf and 1 and 3 days post fertilization (dpf). Scale bars: 500 µm. Arrows donate the yolk sac in WT and mutants (3 dpf) and asterisks donate edema in *zip9*^*-/-*^ larvae (3 dpf). **(C)** Chorion diameter of eggs spawned by *zip9* ^-/-^ and *zip9* ^+/+^ females; n = 40. **(D)** Yolk volume of 3 dpf *zip9* ^-/-^ and *zip9* ^+/+^ larvae; n = 16–18. **(E)** Length of *zip9* ^-/-^ and *zip9* ^+/+^ larvae without exogenous feeding between 3–10 dpf; n = 10–21. **(F)** Incidence of pericardial/yolk sac edema in 6 dpf *zip9* ^-/-^ and *zip9* ^+/+^ larvae; n = 5 clutches. All data represents means ± SEM. Significance was determined by Welch's *t*-test (*, *P* < 0.05; **,* P* < 0.001; ***, *P* < 0.0001).

### Generation of a *zip9*^*-/-*^ zebrafish model

The mutation rate in embryos that were microinjected with the CRISPR-Cas9 system was 71% as determined by heteroduplex mobility assay^20^, and 34% of embryos were carriers of the stop codon cassette. A single male fish heterozygous for the cassette was selected as the founder (F0) and bred to develop a homozygous *zip9*^*-/-*^ strain. Sanger sequencing (DNA Sequencing Facility core, The University of Texas at Austin) of exon 3 from F2 fish homozygous for the stop codon cassette indicated a 64 nucleotide insert made up of the stop codon cassette (35 bp) and additional nucleotides introduced on either side of the cassette (Supplementary Fig. 3A). This insertion produces a premature stop codon that would result in a 95 amino acid protein (Supplementary Fig. 3B) that lacks homologous residue(s) that have been predicted to be involved in zinc transport (human His-155)^21^ and androgen binding (human, Ala-167, Val-241, Met-248 and Leu-249)^22^ activities, if transcribed. Western blot analysis confirmed the absence of ZIP9 expression in *zip9*^*-/-*^ ovaries (Fig. 1C; Supplementary Fig. 1A,B).

**Figure 3:**
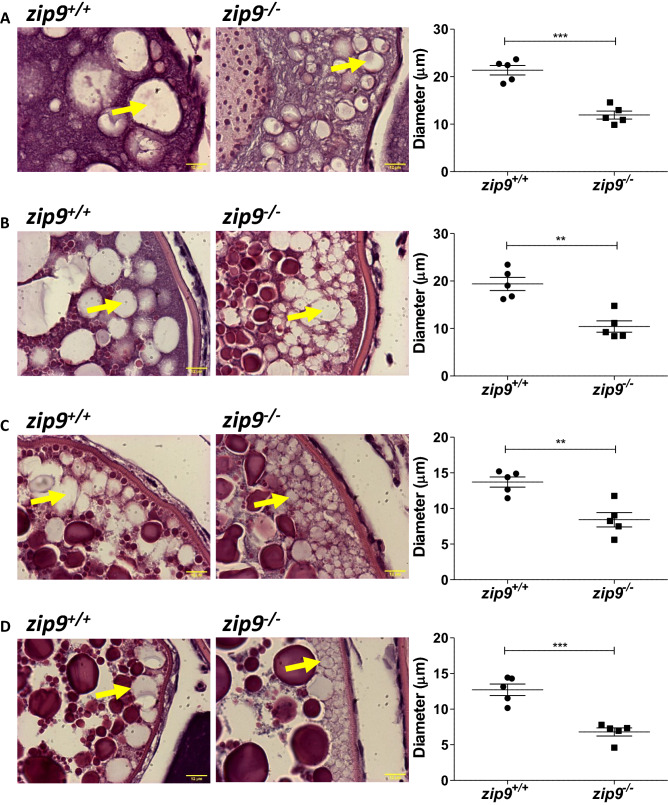
Morphology of cortical vesicles of *zip9*^+*/*+^ and *zip9*^*-/-*^ oocytes throughout oogenesis. Representative images and average cortical vesicle diameters of **(A)** cortical alveoli, **(B)** early vitellogenic, **(C)** mid vitellogenic, and **(D)** late vitellogenic stage *zip9*^+*/*+^ and *zip9*^*-/-*^ oocytes. Arrows indicate location of cortical vesicles. Scale bars: 12 µm. All data represents means ± SEM; n = 5 (average diameter of cortical vesicles for 5 females). Significance was determined by Welch's *t*-test (**, *P* < 0.01; ***, *P* < 0.001).

### *zip9*^*-/-*^ females have reduced reproductive success

Female *zip9*^-/-^ fish showed a significant decrease in spawning incidence (71.41 ± 10.74%) compared to sibling WT controls (100 ± 0.0%) (n = 7/strain; p = 0.021), however *zip9*^*-/-*^ males (90 ± 10.0%) showed no difference to WT (100 ± 0.0%) in breeding occurrence (n = 5/strain; p = 0.375). The number of oocytes spawned per mating event was significantly lower for *zip9*^-/-^ females (127.4 ± 22.06) compared to WT controls (281.7 ± 21.38) (7 females/strain, 2 trials each, n = 14; p < 0.0001). In addition, the percentage of viable *zip9*^-/-^ eggs 2 h post-fertilization (hpf) was less than half that of WT eggs (Fig. 2A, p = 0.0004). There was no significant difference in the fertilization rate of eggs produced by WT females that were mated with *zip9*^-/-^ (97.50 ± 1.26%) or WT (91.79 ± 4.70%) males (5 males/strain, 2 trials each, n = 10; p = 0.279).

### *zip9* mutation disrupts chorion elevation and larval development

**Figure 4:**
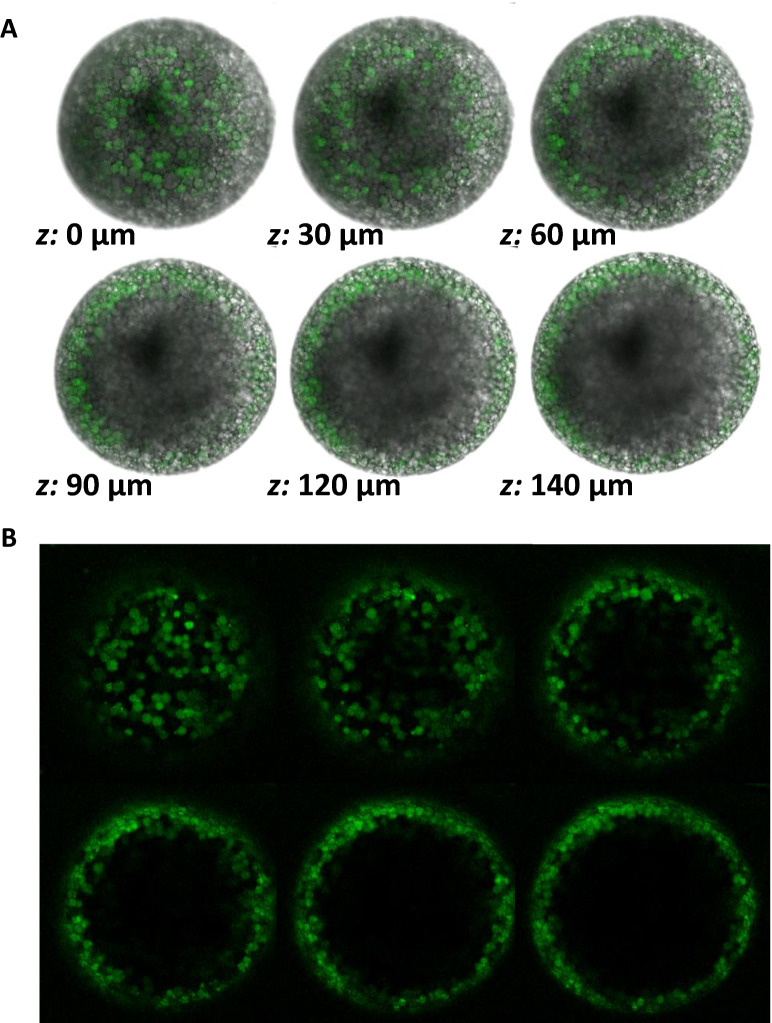
Meiosis II-arrested wildtype zebrafish eggs have cortically-localized, zinc-containing vesicles. **(A, B)** colocalization **(A)** of FluoZin-3-AM signal (green fluorescence) and cortically-located vesicles by brightfield and fluorescent microscopy. Images represent optical slices, with relative location to bottom (*z*: 0) of egg indicated.

Of interest, the majority of *zip9*^-/-^ females produced eggs that did not undergo normal chorion elevation. Only 24.09 ± 11.67% of eggs produced by *zip9*^-/-^ females underwent normal chorion elevation compared to 100% of eggs produced by WT fish (7 females/strain, 2 trials each; n = 14; p < 0.0001). Of the 7 *zip9*^*-/-*^ females used for analysis, only 2 produced eggs which underwent normal chorion elevation (97.15 ± 2.60% and 10.87 ± 13.37% of eggs), thus due to this small sample size, only *zip9*^*-/-*^ eggs with abnormal chorion elevation were included in the subsequent analyses. Eggs from *zip9*^*-/-*^ females had significantly smaller diameters than those produced by WT females (Fig. 2B—4 h post-fertilization (hpf); Fig. 2C, p < 0.0001). Representative images of *zip9*^+*/*+^ and *zip9*^*-/-*^ embryos [4 hpf-3 days post fertilization (dpf)] are presented in Fig. 2B. *zip9*^*-/-*^ embryos had reduced subchorionic space compared to WT embryos (Fig. 2B—4 hpf-1 dpf). At 3 dpf, embryos from *zip9*^*-/-*^ eggs had significantly larger yolk volumes compared to WT embryos (shown by arrows in Fig. 2B—3 dpf; Fig. 2D, p < 0.0001). However, with no exogenous feeding, *zip9*^-/-^ larvae had reduced body lengths compared to WT larvae up to 10 dpf (yolk fully depleted), suggesting that the mutants were unable to utilize yolk stores to obtain growth similar to WT controls (Fig. 2E, p < 0.0001–0.0002). At 6 dpf, *zip9*^-/-^ larvae also showed a four-fold increase in the incidence of edema compared to WT larvae (shown by asterisks in Fig. 2B—3 dpf; Fig. 2F, p = 0.0184).

### Abnormal cortical vesicle development in *zip9*^*-/-*^ eggs

Histological examination of zebrafish ovaries showed that the relative proportion of vitellogenic follicles (early, mid, and late stage) was not significantly different between WT and *zip9*^-/-^ fish (Supplementary Fig. 4A,B). However, *zip9*^*-/-*^ oocytes had cortical vesicles (CVs) with significantly smaller diameters throughout development (cortical alveoli, and early, mid, and late vitellogenic) compared to WT oocytes (CVs designated by arrows in Fig. 3A–D, p = 0.0002–0.0037).

### Activation-induced zinc exocytosis is disrupted in *zip9*^*-/-*^ eggs

**Figure 5:**
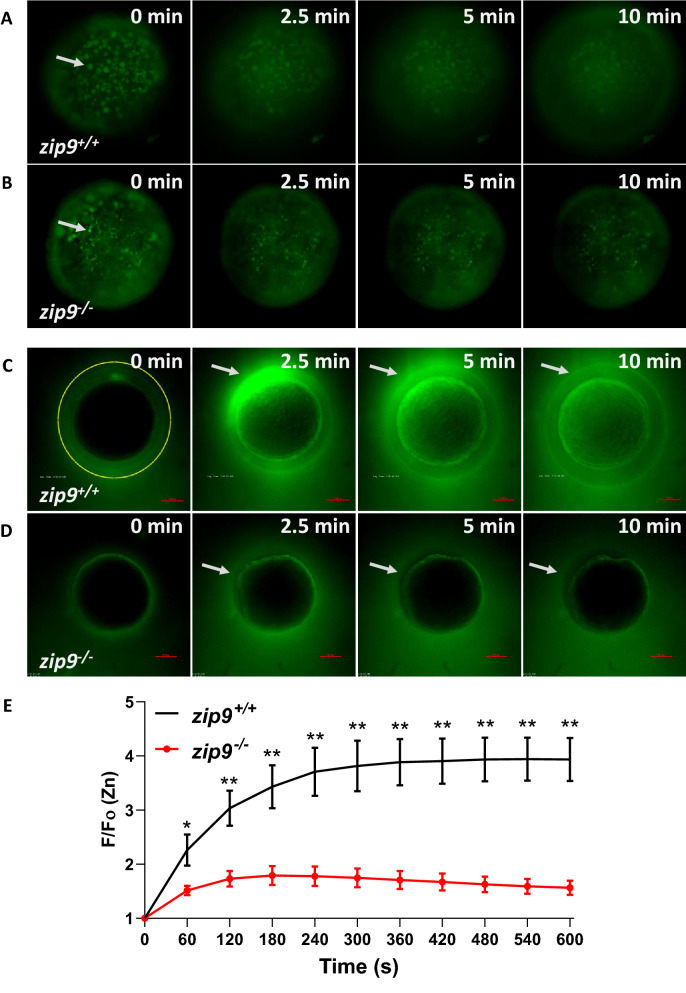
Zinc is stored in cortically-localized vesicles that undergo exocytosis upon egg activation. **(A, B)** *zip9*^+*/*+^ **(A)** and *zip9*^*-/-*^ **(B)** eggs show intracellular zinc staining (arrows, 0 min) in cortically-localized vesicles that are reduced in number at 2.5–10 min post-activation. **(C, D)** Extracellular zinc is observed 2.5–10 min post-activation of *zip9*^+*/*+^ **(C)** and *zip9*^*-/-*^ **(D)** eggs. **(E)** Time trace of normalized extracellular zinc fluorescence by ROI analysis (ROI denoted by yellow circle in **C**, 0 min) 0–10 min post-activation of *zip9*^+*/*+^ and *zip9*^*-/-*^ eggs. Visualization was performed on eggs from a minimum of 3 females/strain. For **(E)**, data represents means ± SEM; n = 5–6. Significance was determined by Welch's *t*-test (*, *P* < 0.05; **, *P* < 0.01).

Visualization of intracellular free zinc in pre-activated WT eggs with the zinc-specific fluorophore FluoZin-3-AM indicated that zinc was localized to discreet cortically-located vesicles (Fig. 4A,B; Fig. 5A, 0 min). The zinc-containing vesicles of *zip9*^*-/-*^ eggs (Fig. 5B, 0 min) were significantly smaller (11.88 ± 0.67 µm, n = 5) than those observed in WT eggs (26.0 ± 1.66 µm, n = 4; p = 0.0043) (Fig. 5A,B; 0 min), consistent with the smaller CVs observed in *zip9*^*-/-*^ oocytes by examination of ovarian histology (Fig. 3A–D). In both WT and *zip9*^*-/-*^ eggs, the cortically-located zinc vesicles were reduced in number upon activation [Fig. 5A,B; 2.5–10 min; Supplemental Movie 1 (WT); Supplemental Movie 2 (*zip9*^*-/-*^)]. To confirm the use of FluoZin-3-AM as a zinc specific indicator in this model, WT eggs loaded with FluoZin-3-AM were treated with the zinc chelator TPEN (10 and 50 µM; N,N,N′,N′-tetrakis(2-pyridinylmethyl)-1,2-ethanediamine). TPEN treatment at both doses resulted in a significant decrease in fluorescence compared to vehicle controls after just 15 min, indicating the observed fluorescent signal is specific to zinc (Supplementary Fig. 5A,B). Detection of extracellular zinc using the non-penetrable zinc fluorophore FluoZin-3 showed zinc was increased after activation in the medium surrounding the eggs of both WT and mutants [Fig. 5C,D; 2.5–10 min; Supplemental Movie 3 (WT); Supplemental Movie 4 (*zip9*^*-/-*^)]. However, the increase in extracellular zinc was significantly greater for WT eggs compared to *zip9*^*-/-*^ eggs between 1–10 min post-activation as determined by quantification of the FluoZin-3 signal (Fig. 5E). Of note, an asymmetrical FluoZin-3 signal was observed during the activation of some eggs (Fig. 5C). Brightfield examination of eggs which displayed asymmetric extracellular zinc release indicated that the zinc signal correlated to the region near the animal pole (Supplementary Fig. 6A,B). The initiation of the calcium wave occurs at the animal pole and drives the cortical reaction in fish species^23,24^. Thus, the initial exocytosis of zinc may occur at the animal pole and become more homologous as the cortical reaction occurs across the egg, but further analysis would be needed to confirm this.

**Figure 6:**
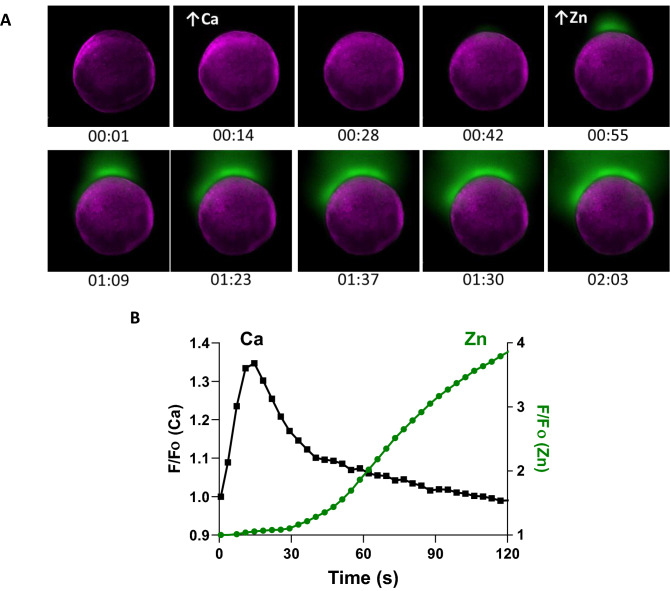
A rise in intracellular calcium proceeds zinc exocytosis in WT eggs. **(A)** Representative time-course images from concurrent intracellular calcium (purple) and extracellular zinc (green) monitoring. **(B)** Representative time trace of normalized calcium (black) and zinc (green) fluorescence signal.

### A calcium rise precedes zinc exocytosis

In mammalian models, zinc sparks correspond with the activation-induced rise of intracellular calcium^14–16^. To determine if there is a correlation between zinc exocytosis and intracellular calcium transients during zebrafish egg activation, both ions were monitored simultaneously (Fig. 6A,B, Supplemental Movie 5). A rise in calcium was observed immediately following activation, after which it steadily decreased to near basal levels by 2 min post-activation. This rapid rise in calcium appeared to be cortically-localized, consistent with prior observations in zebrafish^23,25^. Furthermore, the increase in calcium always preceded the increase in extracellular zinc (zinc fluorescence increased by 10% 35.0 ± 12.4 s post maximal calcium fluorescence, n = 7).

### Pharmacological modulation of zinc affects egg activation

**Figure 7:**
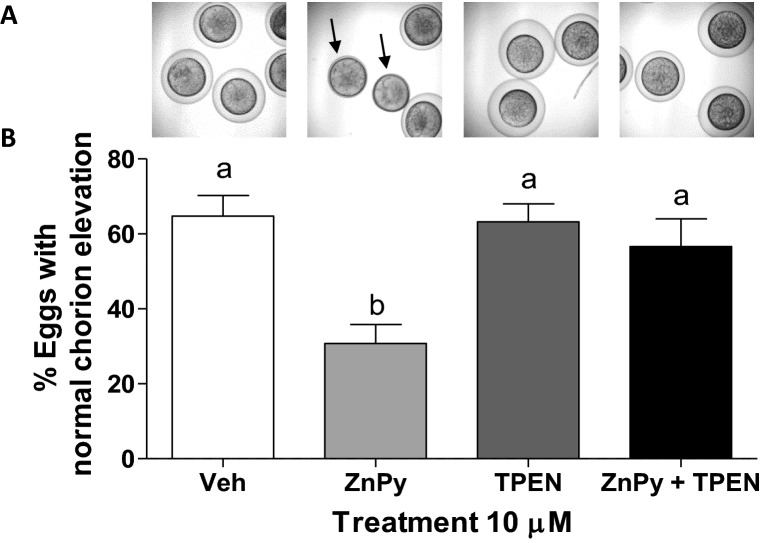
Effect of sustained zinc elevation on chorion elevation. **(A)** Representative micrographs of the effects of pre-treatment with zinc pyrithione (ZnPy) and TPEN on meiosis II-arrested eggs 10 min post-activation. **(B)** Quantification of ZnPy and TPEN-treated eggs that underwent chorion elevation post-activation. All data represents means ± SEM; n = 9–12. Each treatment was repeated in triplicate with similar results obtained for each, and the experiment was repeated with 3–4 fish. Significance was determined by one-way ANOVA with Bonferroni multiple comparison post-test. Different letters indicate significant differences between treatment groups in the post hoc test (*P* < 0.05).

The potential involvement of zinc in chorion elevation of zebrafish eggs was investigated by pharmacological elevation of intracellular zinc levels with a zinc ionophore, zinc pyrithione (ZnPy). Treatment of WT meiosis II-arrested (pre-activated) eggs with ZnPy resulted in a significant decrease in the percent that underwent normal chorion elevation when compared to eggs that were treated with vehicle control (Fig. 7A,B). Co-treatment with the intracellular zinc chelator TPEN attenuated the response to ZnPy, which resulted in eggs that underwent normal chorion elevation similar to controls, while TPEN alone had no effect. This suggests that reduction of intracellular zinc is essential to proper egg activation in teleosts, a finding similar to that observed in mice in which ZnPy treatment maintains eggs in a meiosis II-like state after activation is induced^26^.

## Discussion

Here we present the first evidence for a role of the zinc transporter/membrane androgen receptor ZIP9 in the reproductive success of a female vertebrate. *zip9*-mutants show reduced fecundity, egg viability, and reduced larval growth and survival. *zip9*^*-/-*^ females produce a high proportion of eggs that undergo abnormal chorion elevation, which coincides with abnormalities in cortical vesicle morphology throughout oogenesis. Furthermore, this study demonstrates that zinc is stored in cortically-located vesicles in meiosis II-arrested zebrafish eggs. This zinc is exocytosed during activation, as demonstrated by the loss of intracellular zinc vesicles and a corresponding increase in zinc in the extracellular milieu. These events are similar to those observed in mammalian models in which zinc has been shown to be tightly regulated during oocyte maturation and egg activation. Of interest, *zip9*^*-/-*^ eggs that do not undergo chorion elevation have smaller zinc-containing vesicles and a significant reduction in the amount of zinc exocytosed during activation compared to WT eggs. *zip9* mutation results in abnormal zinc regulation in the egg during development and a corresponding altered activation response which culminates in a severe reduction in reproductive success.

While the role of zinc in mammalian egg activation has been extensively investigated over the past decade, few studies have examined the role of zinc in eggs of non-mammalian vertebrates. In mammalian models, the meiosis I—meiosis II transition has been shown to coincide with an increase in oocyte zinc content^13,27^. Furthermore, meiosis II resumption is associated with zinc exocytosis, or a zinc “spark,” following fertilization-induced activation^14–16,28^. This zinc release has recently been shown to require myosin light chain kinase activity (MLCK)^29^, which is essential to proper cortical vesicular exocytosis^30,31^. This supports the proposal that zinc is stored in cortical vesicles and released upon initiation of the cortical reaction. In the current study, we demonstrate that in pre-activated zebrafish eggs (meiosis II-arrested), zinc is stored in cortically-located vesicles that undergo exocytosis upon activation. This response is unmistakably similar to that of the zinc “spark” observed in mammals. Zinc exocytosis was found to occur after an initial rise in calcium, which suggests these two events may be correlated in zebrafish as they are in mammalian species^14–16^. In addition, sustained zinc elevation by treatment with ZnPy prevented activation-induced chorion elevation, which suggests that zinc modulation can regulate this event in fish. While further work is required to confirm the exact roles of zinc in egg activation in zebrafish, we present the first evidence of zinc modulation during egg activation in a non-mammalian model.

Differences in the characteristics of the zinc exocytosis of zebrafish eggs compared to those of mammalian zinc sparks likely results from physiological, size, and procedural differences. While in mammals, zinc sparks peak and decrease within the time-frame of a minute^14–16^, most WT zebrafish eggs exhibited a sustained extracellular zinc signal after an initial rapid rise. Only 33% of WT eggs showed a clear peak and decline. Conversely, all *zip9*^*-/-*^ eggs exhibited peaks within 5 min of activation, and then showed a decline in signal. The lack of peaks in the zinc signal from WT eggs likely results in partial oversaturation of the activation media droplet by zinc-bound FluoZin-3, due to the large size of the eggs. Furthermore, in the eggs that did exhibit a peak and decline in zinc signal, the timeframe to reach peak fluorescence intensity (175.3 ± 16.8 s post-activation) was longer than that observed in mammals, indicating that zinc exocytosis likely occurs over a longer time period in zebrafish.

In the current study, *zip9* mutation produces an abnormal activation phenotype resulting in decreased egg and larval viability. The decrease in larval viability and growth is likely driven by the limited subchorionic space in *zip9*^*-/-*^ eggs, while the reduced egg viability at 2 hpf is indicative of poor egg quality. In mammals, meiosis is tightly regulated by zinc and zinc-insufficient oocytes fail to reach metaphase II arrest^13,14^. If zebrafish *zip9*^*-/-*^ eggs have insufficient zinc levels, this could similarly disrupt meiotic maturation. Decreased egg viability may also result from an inability of the chorion to prevent polyspermy, which is lethal. In zebrafish, the cortical reaction begins shortly after the egg is released and establishes various mechanical barriers that prevent sperm access through the micropyle. These include the elevation of the chorion, which separates the micropyle from the fertilization cone, and the exocytosis of cortical vesicles that contain factors that immobilize sperm and function to harden the chorion^24,32^. In murine eggs, zinc is accumulated in the zona pellucida upon activation, coincident with the zinc spark, and causes physiochemical changes that reduce sperm binding^33^. The lack of chorion elevation as well as the smaller cortical vesicles of *zip9*^*-/-*^ eggs may indicate that they are deficient in the composition or quantity of constituents, such as zinc, essential to chorion elevation and that potentially aid in the prevention of polyspermy. While it remains unclear how the disruption of zinc dynamics results in decreased viability in *zip9*^*-/-*^ zebrafish, these findings are consistent with the observation that the intensity of the zinc spark correlates to embryo quality in mammals^34^. Murine zygotes that develop into blastocysts release more zinc upon activation than those that fail early in development^34^, and similarly, in the current study, *zip9*^*-/-*^ eggs have a dampened zinc release and show a significant decrease in viability.

Members of the SLC39A (ZIP) family mediate zinc transport from outside the cell and from intracellular stores into the cytoplasmic compartment, while members of the SLC30 (ZnT) family mediate zinc transport out of the cytoplasm. In zebrafish, the high ZIP9 expression on the plasma membrane of early/mid stage vitellogenic oocytes and ovulated eggs indicate ZIP9 most likely transports extracellular zinc into oocytes and eggs. The abnormal morphology of zinc-containing cortical vesicles and decreased zinc exocytosis exhibited by *zip9*-mutants supports this. However, there are likely other zinc transporters (ZnT members) involved in the packaging of zinc into cortical vesicles, since SLC39A members cannot move zinc into intracellular compartments. In mammals, oocytes accumulate zinc during maturation^13^, and the SLC39A members ZIP6 and ZIP10 have been shown to be vital for zinc regulation during prophase I arrest and meiotic maturation in mice^28^. However, it remains unknown if SLC39A members play a role in the acquisition of zinc that is released during the zinc spark in mammalian models. It is possible that in zebrafish ZIP9 plays a role in the acquisition of zinc into the egg prior to the resumption of meiosis since ZIP9 is highly expressed on the plasma membrane in early and mid stage vitellogenic oocytes. This zinc acquisition may then be essential to a zinc exocytosis response that is required for meiosis resumption and/or zona pellucida hardening, similar to that observed in mammalian models. Additional examination of the role of zinc and ZIP9 in these processes would be required to confirm this. Thus, the stage of oogenesis and the mechanism by which ZIP9 is involved in zebrafish egg zinc dynamics remains unclear at present.

The phenotype of the *zip9*^*-/-*^ eggs is remarkably similar to that observed by Mei et al. 2009 in heterogeneous nuclear ribonucleoprotein I (hnRNP I) mutant zebrafish^35^. hnRNP I mutants are defective in the activation-induced IP3-mediated rise in Ca^++^, which results in failed cortical vesicle exocytosis and abnormal chorion elevation. The similarities in this phenotype to that of the *zip9*^*-/-*^ eggs indicate that both of these proteins are essential for proper egg activation in zebrafish. Furthermore, these studies highlight the essentiality of proper cortical vesicle exocytosis to chorion elevation, with both complete failure (hnRNP I) of exocytosis as well as abnormal cortical vesicle development (ZIP9) resulting in similar phenotypes.

It is important to note the rapid rise in intracellular Ca^++^ during egg activation occurs in all vertebrate (*Xenopus*^36^; teleosts^25,37^; mammals^38,39^) and invertebrate (sea urchin^40^; *Drosophila*^41^; *C. elegans*^42^) models examined to date, which make it the most conserved and identifying feature of egg activation. To date, zinc’s role in activation has only been examined in a limited number of mammalian models as well as zebrafish in the current study. It remains unclear how similar zinc’s role is between teleost and mammalian models and if other phyla also utilize zinc modulation during egg activation. However, the highly conserved nature of the calcium wave during activation, as well as the recent understanding that zinc is an important signaling factor that acts similarly to calcium in various tissue and cell models^43,44^, highlights the need for a comparative examination of the role of zinc in oocyte development and maturation.

While this study exhibits the role of ZIP9 in egg zinc dynamics, it is unclear whether the androgen receptor activity of ZIP9 is involved in modulation of zinc. ZIP9 is known to have the capacity to concurrently act as an androgen receptor and zinc transporter in several cell models^1–3^, while in other cells the protein’s zinc transporter activity can function in the absence of androgen stimulation^8–10^. In the current model, the possibility that androgens play a role in the zinc dynamics mediated by ZIP9 remains to be investigated. Although the oocyte stage and specific mechanism by which ZIP9 acts to mediate zinc in this model is unknown at present, high concentrations of androgens are present in teleost ovaries throughout oogenesis^45^. Androgens have also been shown to promote folliculogenesis in both fish and mammalian models^46–51^. Thus, androgens have the potential to mediate zinc transport through ZIP9 at least during certain stages of ovarian follicle and oocyte development, which will be the subject of a future investigation.

In conclusion, ZIP9 (Slc39a9) plays a crucial role in zebrafish egg zinc dynamics. Here we present the first evidence of a zinc spark in a teleost model, however, further examination of zinc throughout the oocyte-to-egg and egg-to-embryo transitions is required to conclude how similar zinc modulation is between teleost and mammalian models. Furthermore, while other SLC39A members have been found to mediate mammalian oocytes zinc dynamics, it remains unknown if ZIP9 can also modulate zinc in mammalian oocytes as well. Altogether, this work highlights the need for a comparative examination of the role of zinc in oocyte and egg physiology as well as the role of zinc transporters in the regulation of zinc-dependent meiotic events.

## Materials and methods

### Zebrafish husbandry

Wildtype ZDR strain zebrafish (*Danio rerio*) obtained from Segrest Farms (Gibsonton, FL, USA) were maintained in a 14 h light:10 h dark cycle at 28.5 °C at the University of Texas Marine Science Institute in Port Aransas, TX. Adult fish were fed to satiation 1–2 times/day. Early stage larval fish (5 dpf-10 dpf) and later stage larval fish (10 + dpf) were fed twice daily with boiled egg yolk and *Artemia*, respectively. For egg and tissue collection, fish were deeply anesthetized by immersion bath in 150 mg/L (egg collection) or 300 mg/L (lethal) buffered MS-222, respectively. Fish were humanely euthanized by rapid decapitation. Procedures were approved and carried out in accordance with the ethical guidelines and regulations of the University of Texas Animal Care and Use Committee (protocol: AUP-2019-00229).

### RNA isolation and quantitative real-time PCR analysis

Follicles of different stages [early-mid vitellogenic (400–550 µm) and full grown (> 600 µm), and ovulated eggs (meiosis II-arrested)] were isolated from WT zebrafish ovaries, after which the follicles were treated with 0.01% collagenase in 60% L-15 (Leibovitz's) media for 30 min to remove the follicle cells. After digestion the follicles were repeatedly pipetted, and the follicle cells were collected by centrifugation of the media at 1,500 *g* for 5 min. The oocytes were repeatedly rinsed with 60% L-15 and the complete removal of follicle cells was confirmed by DAPI staining of a subset of oocytes. Total mRNA was isolated from oocytes and follicle cells using Tri Reagent (Sigma-Aldrich, St. Louis, MO) following the manufacturer’s protocol. Quantitative real-time PCR (qPCR) primers are listed in Supplementary Table 1. qPCR was performed using Verso 1-step RTqPCR SYBR Green Low ROX kit (Thermo Scientific, Waltham, MA), with 50 ng of mRNA per 15 μL reaction, following the manufacturer’s protocol. The qPCR program was as follows, 50 °C for 18 min, 95 °C for 15 min, and 35 cycles of 95 °C for 15 s, 55 °C for 30 s, and 72 °C for 30 s. Amplification was followed by the melting curve program, 95 °C for 30 s, 60 °C for 15 s and a gradual increase to 95 °C over 20 min. Samples were run in duplicate and expression of *zip9* was normalized to *18 s*.

### Antibody production

Zebrafish ZIP9 antigen peptide and antibody synthesis and purification were performed by GenScript (Piscataway, NJ). A peptide of the amino acid sequence HSHSPGGSAGKGLS was synthesized with a cysteine residue on the N-terminus to facilitate conjugation of keyhole limpet hemocyanin (KLH) and used for polyclonal antibody production in rabbits.

### Western blot analysis

Plasma membrane protein preparation and Western blot analyses were performed following procedures outlined in Converse & Thomas 2019. Briefly, oocytes of the same stage were pooled from 2–3 fish and denuded of follicle cells before membrane protein preparation. Ovulated eggs were not pooled between fish. After electrophoresis and transfer, the nitrocellulose membrane was incubated with antibodies targeting ZIP9 (1 µg/ml) or actin (1:1,000; Thermo Scientific, MS-1295-P0) overnight at 4 °C. The following day, the membrane was washed in phosphate buffered saline (PBS) (NaCl 137 mM, KCl, 2.7 mM, Na2HPO4 10 mM, KH2PO4 1.8 mM) followed by incubation with the secondary antibodies (1:15,000; LI-COR IRDye 800CW goat anti-rabbit 800CW; LI-COR IRDye 680RD goat anti-mouse; LI-COR, Lincoln, NE) for 1 h at room temperature. Protein bands were visualized using a ChemiDoc Imaging System (Bio-Rad Laboratories, Hercules, CA) and analyzed using ImageJ (NIH, Bethesda, MD). The specificity of the ZIP9 antibody was verified by incubating the antibody solution with excess antigen peptide (20 × molar concentration) overnight at 4 °C prior to use in Western blot analysis, which confirmed the absence of signal (Supplementary Fig. 1).

### Design of *zip9*-targeted CRISPR-Cas9 system and gRNA preparation

Targets for *zip9* mutagenesis were determined using CRISPOR (https://crispor.tefor.net/). Design and generation of guide RNA (gRNA) and a stop codon cassette was performed using the methods described in the supplemental protocol for Gagnon et al. 2014^52^. A target site in exon 3 was chosen due to its high efficiency and low off-target scores. gRNA specific to this site was generated using a MAXIscript Kit (Invitrogen, Waltham, MA), DNase treated, purified by ethanol precipitation, verified for correct length on an agarose gel, and stored at − 80 °C until use. All oligonucleotides were purchased from Invitrogen and are listed in Supplementary Table 2.

### Establishment of zebrafish *zip9*-mutant strain

To generate global *zip9*-mutants, an injection mixture containing 0.5 µl 1× Cas9 NLS (New England Biolabs, Ipswich, MA), 0.3 µl gRNA (1 µg/µl), 0.3 µl stop codon cassette oligonucleotide (3 µM), 0.25 µl phenol red, and 1.32 µl nuclease-free water was incubated for 5 min at room temperature and then stored on ice until use in embryo microinjection. WT eggs were collected within 30 min of fertilization, and 2 nl of injection mixture was injected into the one-cell stage embryo using a micromanipulator (Narishige, Amityville, NY) and microinjector (Tritech Research, Inc., Los Angeles, CA). DNA was isolated from fish after 4 dpf using the HotSHOT method^53^, and PCR was used to screen for the presence of the stop codon cassette. Primers used for screening are listed in Supplementary Table 3. A male founder (F0) heterozygous for the knock-in of the stop codon cassette was raised to adulthood and mated with WT females to obtain F1 offspring which were bred to obtain a *zip9*^*-/-*^ homozygous strain.

### Fecundity analyses

Four month old F2 *zip9*^+*/*+^ and *zip9*^*-/-*^ fish were used for fecundity measures. *zip9*^+/+^ and *zip9*^-/-^ fish were left undisturbed with breeding-confirmed WT fish in breeding tanks for 2 h after lights on. The fish were then removed from the tanks and verification of spawning and egg analyses were performed. Each fish was spawned twice with a 4-day rest between events. Spawning incidence was determined as the percentage of spawning in two independent events for each individual [(number of times spawned/2) × 100].

### Embryo and larval assessments

Growth of embryo and larval fish was assessed on F3 *zip9*^+*/*+^ and *zip9*^*-/-*^ fish. *zip9*^*-/-*^ embryos from eggs with normal and abnormal chorion elevation were separated before hatching so that the growth and incidence of edema could be assessed for the two phenotypes separately. For analyses, fish were imaged eye-over-eye in 3% methylcellulose (Sigma-Aldrich).

### Histological analyses

Histological analyses were performed on 3–6 month old, breeding-confirmed, F2/F3 *zip9*^+*/*+^ and *zip9*^*-/-*^ females. Ovarian tissue was fixed in 4% paraformaldehyde for 3–4 days, and histological preparations were performed by Pacific Pathology, Inc. (San Diego, CA). Ovarian follicle stage identification was performed by referencing prior works^54,55^. Late vitellogenic follicles ranged from 536–644 µm, mid vitellogenic follicles from 420–460 µm, early vitellogenic follicles from 305–342 µm, and cortical alveoli stage from 170–225 µm.

### Fluorescence microscopy of zebrafish egg activation

F2/F3 females were anesthetized prior to stripping for mature eggs which were kept in a pre-activated state (meiosis II arrest) by placing in pre-activation buffer (Hank’s saline: 0.137 M NaCl, 5.4 mM KCl, 0.25 mM Na_2_HPO_4_, 0.44 mM KH_2_PO_4_, 1.3 mM CaCl_2_, 1.0 mM MgSO_4_, 4.2 mM NaHCO_3_; osmolality ~ 290 mOsm/kg) containing 0.5% BSA^56^. Activation of eggs was induced by submersion in either pure water (ASTM type II; to decrease background fluorescence when using zinc fluorophores) or in 0.5× E2 media (7.5 mM NaCl, 0.25 mM KCl, 0.5 mM MgSO_4_, 75 µM KH_2_PO_4_, 25 µM Na_2_HPO_4_, 0.5 mM CaCl_2_, 0.35 mM NaHCO_3_, 05 mg/L methylene blue; osmolality ~ 28 mOsm/kg) for activation assays. For intracellular zinc examination, eggs were incubated at 37 °C in pre-activation buffer with 50 µM FluoZin-3 AM for 2–4 h and then washed with pre-activation buffer. Eggs were placed on a petri dish, excess buffer was wicked away, and a 15 µl drop of water was placed on the egg. Time-series imaging was initiated after the addition of the water droplet using a Nikon Eclipse Ti2 microscope (Nikon Instruments Inc., Melville, NY). Confocal microscopy was performed using a Nikon C2 confocal microscope system with a Nikon Eclipse Ti2-E inverted microscope. For extracellular zinc examination, pre-activated eggs were placed on a petri dish for imaging, excess buffer was wicked away, and a 15 µl drop of 50 µM FluoZin-3 (diluted in water) was added to the egg and time-series imaging was initiated. For concurrent intracellular calcium and extracellular zinc examination, eggs were first incubated with 2 µM Fura 2-AM (Sigma-Aldrich) and 0.04% Pluronic F-127 (AAT Bioquest, Inc., Sunnyvale, CA) for 1 h at 28 °C, washed in pre-activation buffer, then processed as outlined for extracellular zinc examination. Calcium analysis was performed on 7 eggs from different WT females. Intracellular calcium and extracellular zinc were analyzed by defining regions of interest (ROIs) in which fluorescence intensity was measured using the Time Series Analyzer V3 plugin for ImageJ. The area of extracellular ROIs (zinc analyses) was maintained between analyses and encompassed the egg and the area immediately surrounding the egg. Intracellular ROIs were defined as the total area of the egg.

### Activation assay

15–30 pre-activated WT eggs were placed into wells of a 12-well plate with 1 ml of pre-activation buffer with either vehicle (0.1% DMSO), the zinc chelator TPEN (10 µM; N,N,N′,N′-tetrakis(2-pyridinylmethyl)-1,2-ethanediamine, Caymen Chemical, Ann Arbor, MI), or the zinc ionophore zinc pyrithione (ZnPy; 10 µM; 2-Mercaptopyridine N-oxide sodium salt hydrate, Alfa Aesar, Haverhill, MA) and incubated at 24 °C for 4 h, after which the buffer was removed and 1 ml of 0.5× E2 media was added. The eggs were examined after 10 min for the number that had undergone normal chorion elevation, which was presented as the percent of total eggs/well.

### Statistical analysis

Statistical significance was determined by Welch’s *t*-test or one-way ANOVA with a post hoc Bonferroni multiple comparison test. All data are expressed as the mean ± SEM using Prism 5.0 software (GraphPad Software, San Diego, CA).

## Supplementary information

Supplementary Video 1.

Supplementary Video 2.

Supplementary Video 3.

Supplementary Video 4.

Supplementary Video 5.

Supplementary Information.

## Data Availability

All data generated during this study are available from the corresponding author upon reasonable request.
