# Supplementary material for: The zinc transporter ZIP9 (Slc39a9) regulates zinc dynamics essential to egg activation in zebrafish
Source: Sci Rep. 2020 Sep 24;10:15673. doi: 10.1038/s41598-020-72515-4 (PMC7518430; doi:10.1038/s41598-020-72515-4)
Supplement: Supplementary file 6 — Supplementary Information. [file 41598_2020_72515_MOESM6_ESM.pdf]

Supplementary Materials

The zinc transporter ZIP9 (Slc39a9) regulates zinc dynamics essential to egg activation in zebrafish

Aubrey Converse[1], Peter Thomas[1]

[1]Marine Science Institute, The University of Texas at Austin, Port Aransas, Texas, U.S.A

Supplementary Table 1

Primers used for qPCR.

Primer	Forward	Reverse
zip9	AGCAGCTCCACGCTTACATT	CATGGACGACCAATCCGAGT
18s	AGAAACGGCTACCACATCCA	TCCCGAGATCCAACTACGAG

Supplementary Table 2

Oligonucleotide sequences used in gRNA generation and stop codon cassette.

Primer	(5'-3')
**Exon 3 specific**	TAATACGACTCACTATAGAAGGAGTGGTGAGACCCAGGTTTTAGAGCTAGAAATAGCAAG
**tracrRNA tail**	AAAAGCACCGACTCGGTGCCACTTTTTCAAGTTGATAACGGACTAGCCTTATTTTAACTTGCTATTTCTAGCTCTAAAAC
**Exon 3 specific stop codon cassette**	GCAGAAGGAGTGGTGAGACCGTCATGGCGTTTAAACCTTAATTAAGCTGTTGTAGCAGCGGTGAACACGGCCACG

Supplementary Table 3

Primers used in screening assays.

Primer	Forward	Reverse
**Heteroduplex mobility assay**	ATGCATTGTGCTGCTAGTTGTT	CCAGAGAAATCCCAATGTAAGC
**Stop cassette specific**	GGTGAGACCGTCATGGCGTTTA	CGCAGTGTGAGCCAATCCAAATA
**Homo/heterozygous screening**	ATGCATTGTGCTGCTAGTTGTT	CGCAGTGTGAGCCAATCCAAATA

Supplementary Figure 1

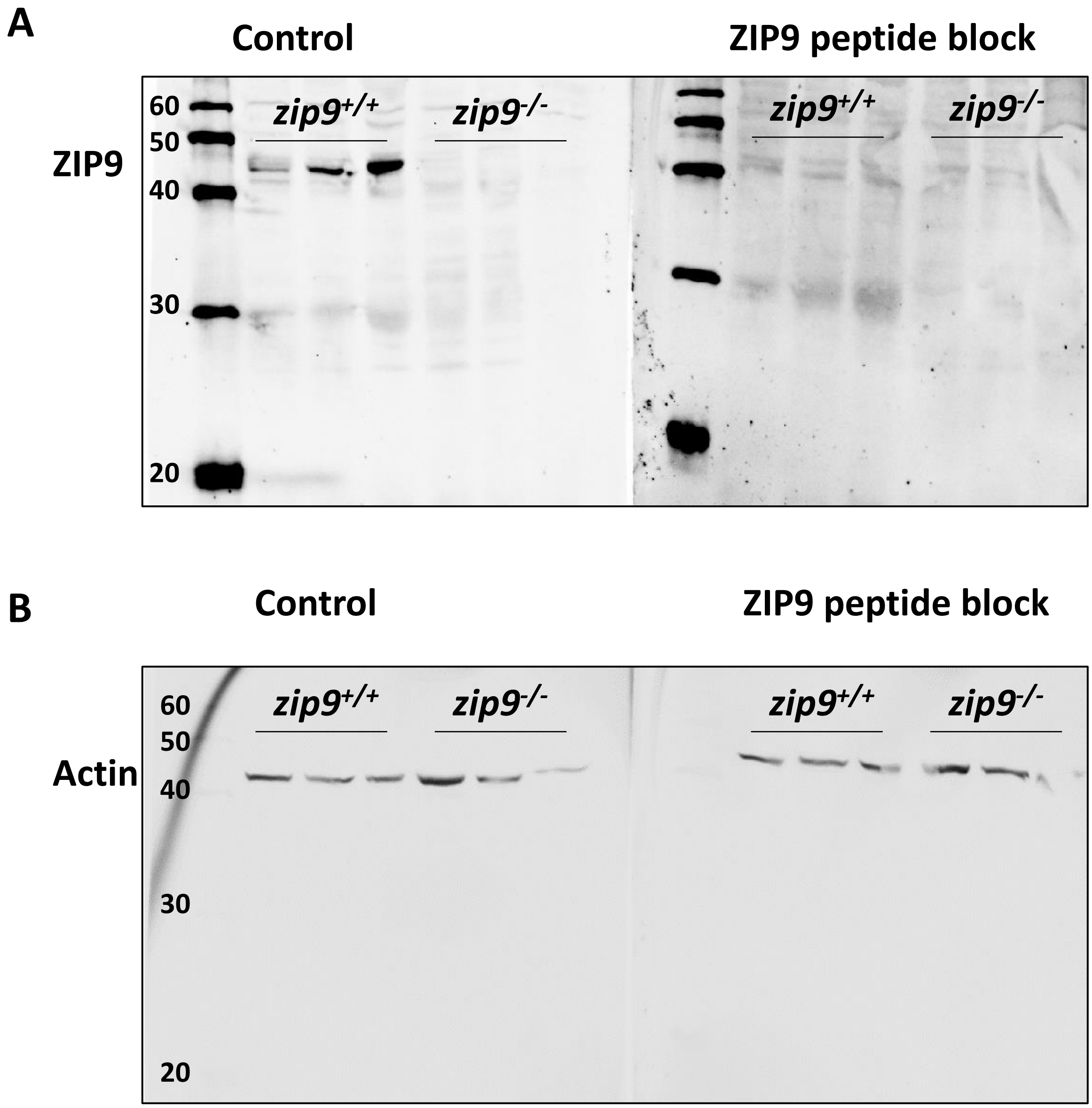

Supplementary Figure 1. Zebrafish ZIP9 antibody validation. A-B, Western blots of plasma membrane fractions (5 µg) from *zip9* $^{+/+}$ and *zip9* $^{-/-}$ ovaries incubated with control or peptide blocked ZIP9 antibody (A), or actin housekeeping control (B).

Supplementary Figure 2

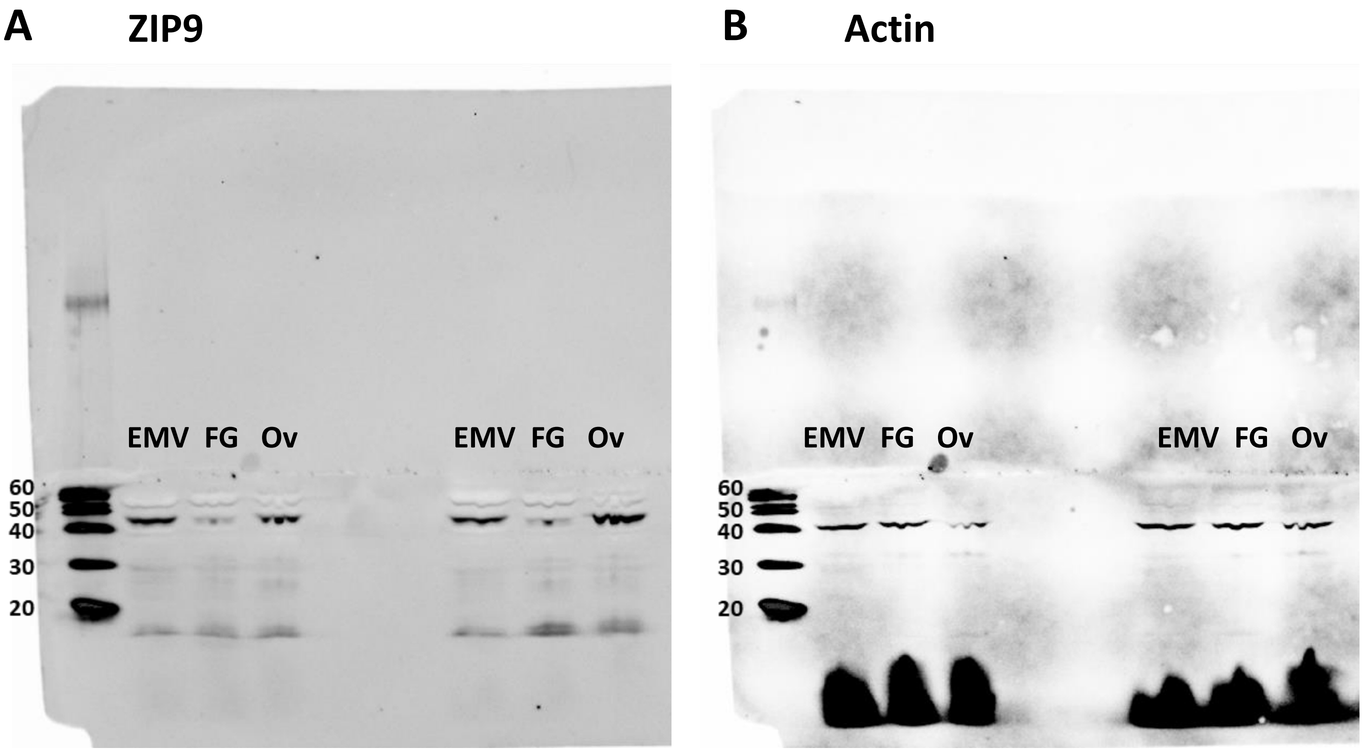

Supplementary Figure 2. Full blots of Figure 1B; ZIP9 protein expression on plasma membrane of different stage oocytes/eggs. A, Detection of ZIP9 antibody. B, Detection of Actin antibody. Two representative samples of each stage shown. EMV, early-mid vitellogenic oocytes; FG, full grown oocytes; Ov, ovulated eggs.

Supplementary Figure 3

A

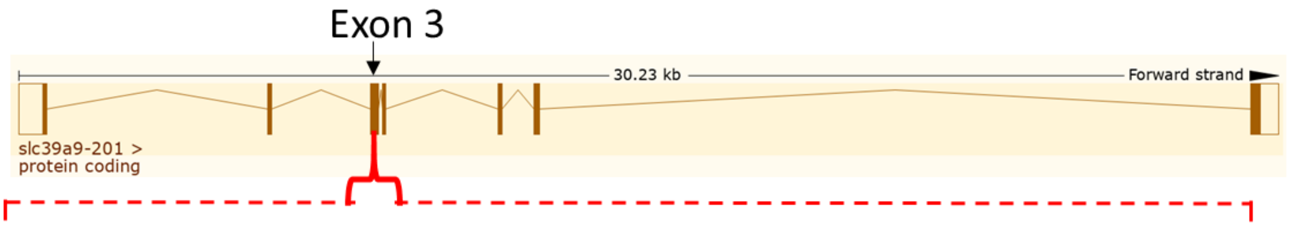


```
zip9+/+ 5'CAGAAGGAGTGGTGAGACCC-------------------------------------AGC-------------------------GGTGAACACGGCCACGG 3'
          |||||||||||||||||||                                    |||                          ||||||||||||||||| 
zip9-/-   CAGAAGGAGTGGTGAGACCCTTGTAAACGCCATGTCATGGCGTTTAAACCTTAATTAAGCTGTTGTAGATGAGGAAATGCTGGAAGGTGAACACGGCCACGG
```

B

ZIP9$^{+/+}$

M D D F S S I S L L S L S M L I G C Y V A G T I P L A V N
F S E E K L K L V T V L G A G L L C G T A L A V I I P E G
V H A L Y E E M L E G V H N H G H G Q V E A E V S E Q
K V A E G V V R P S G E H G H G H E Q L H A Y I G I S L
V L G F V F M L L V D Q I G S A H M H S S D D P E A A
R A A S S K I T T T L G L V V H A A A D G V A L G A A A S
T S Q T S V Q L I V F V A I M L H K A P A A F G L V S F
L M H A G L E R N R I R K H L L V F A L A A P V L A M V T
Y V G L S Q S S K E A L S D V N A T G V A M L F S A G T
F L Y V A T V H V L P E V G G M G G H S H S P G G S A
G K G L S K L E V G A L V L G C L I P L V L S I G H Q H

ZIP9$^{-/-}$

M D D F S S I S L L S L S M L I G C Y V A G T I P L A V N
F S E E K L K L V T V L G A G L L C G T A L A V I I P E G
V H A L Y E E M L E G V H N H G H G Q V E A E V S E Q
K V A E G V V R P L **Stop**

Supplementary Figure 3. *zip9* mutation generation. A, Schematic representation of *zip9*, and the comparison of mutant *zip9*$^{-/-}$ and *zip9*$^{+/+}$ exon 3 nucleotide sequence. B, Comparison of predicted ZIP9$^{-/-}$ and ZIP9$^{+/+}$ protein sequence. Highlighted nucleotide sequence designates the stop codon cassette, red-text nucleotides indicate stop codons, asterisk indicates stop codon resulting in truncation, red boxed-amino acids are adjacent to stop codon in ZIP9$^{-/-}$.

Supplementary Figure 4

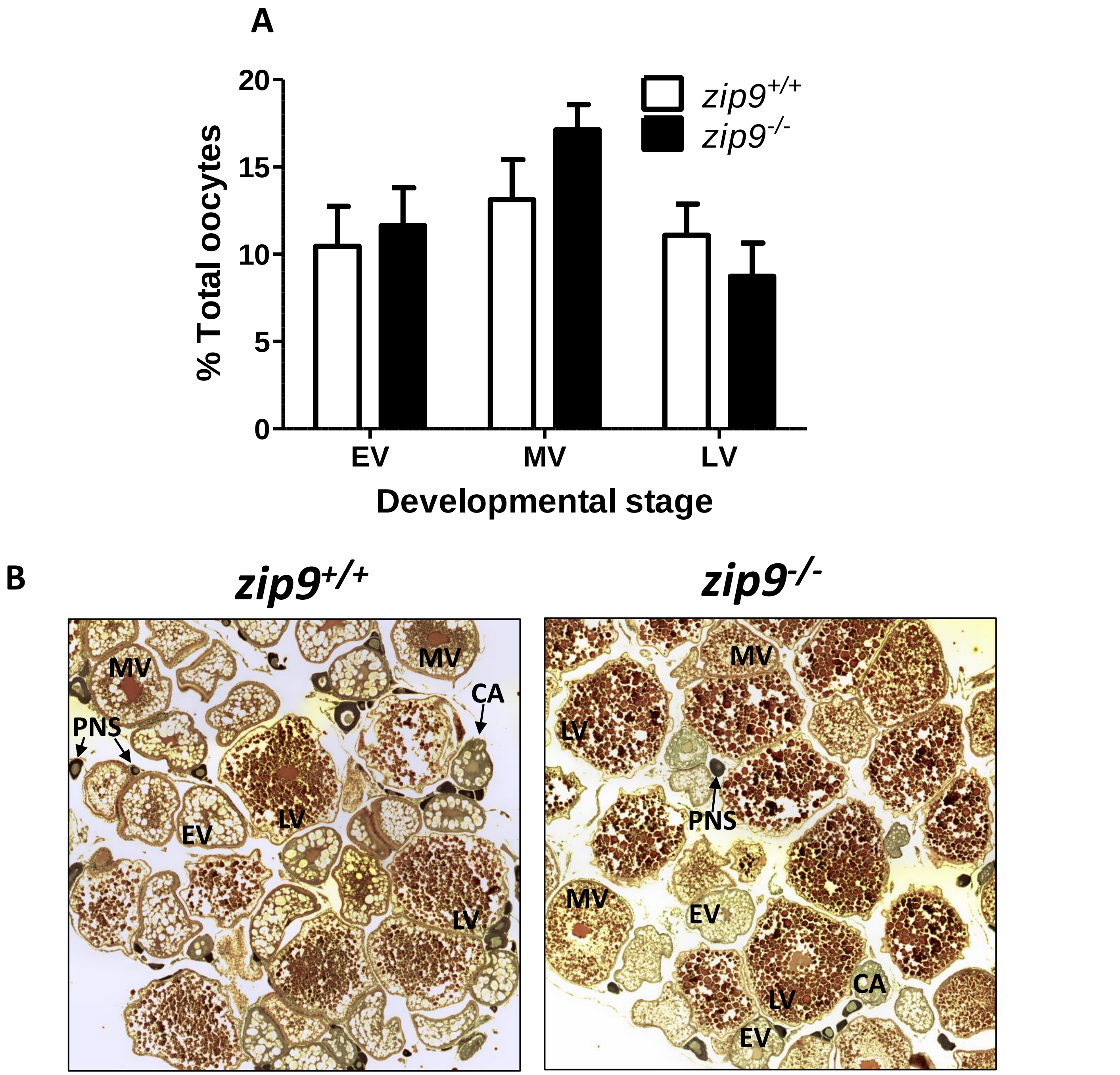

Supplementary Figure 4. Ovarian morphology of *zip9*$^{-/-}$ and *zip9*$^{+/+}$ fish. A, The relative proportion of ovarian follicles at different stages of development in *zip9*$^{-/-}$ and *zip9*$^{+/+}$ ovaries. B, Representative image of ovarian histology of *zip9*$^{-/-}$ and *zip9*$^{+/+}$ fish. All data represents means ± SEM, n=5. PNS, perinuclear stage; CA, cortical alveoli stage; EV, early vitellogenic stage; MV, mid vitellogenic stage; LV, late vitellogenic stage.

Supplementary Figure 5

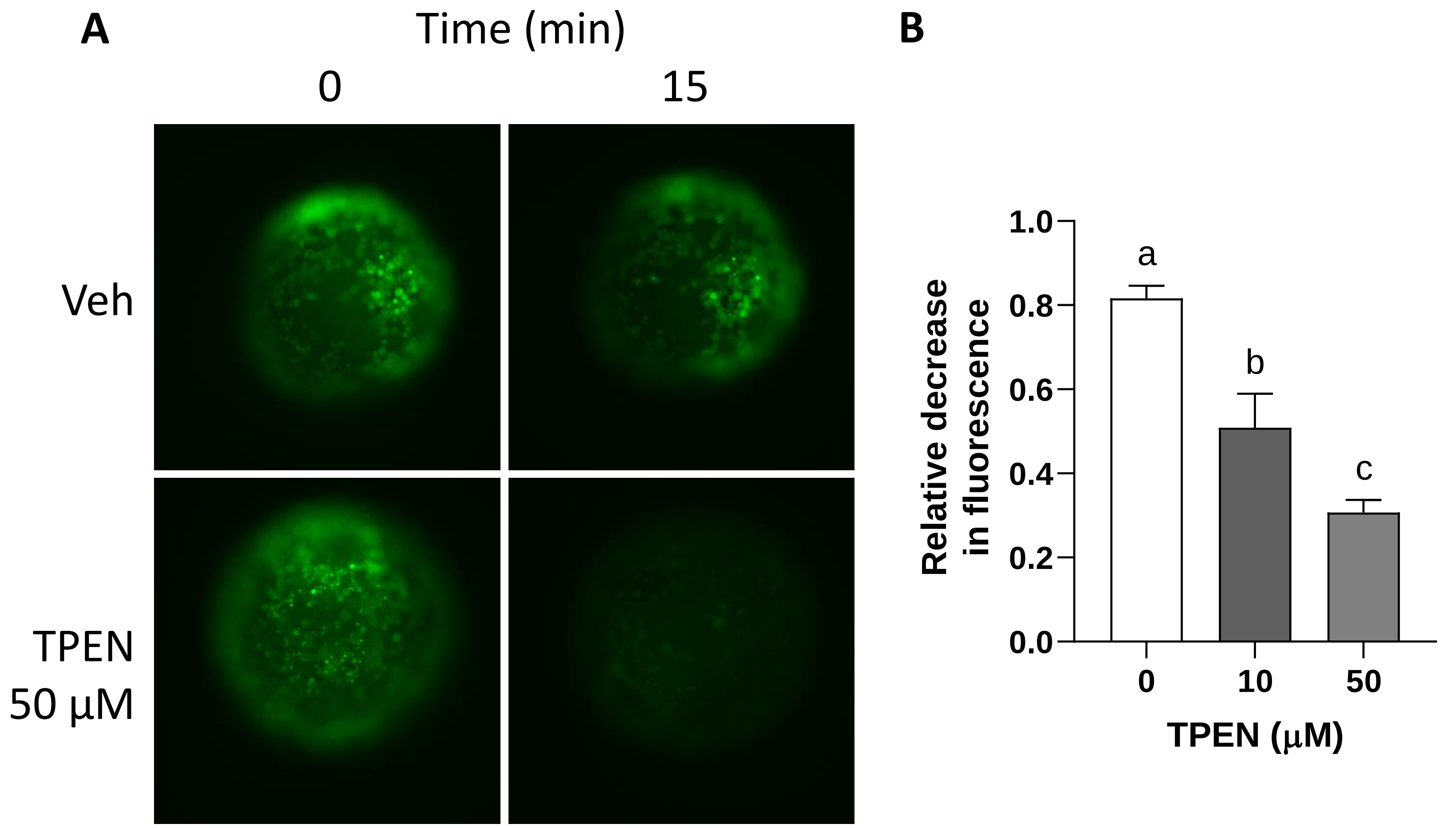

Supplementary Figure 5. Specificity of FluoZin-3-AM. A, Representative images of pre-activated WT eggs loaded with FluoZin-3-AM and treated with vehicle or TPEN. B, Quantification of relative decrease of FluoZin-3-AM fluorescence in TPEN treated eggs. Data represents means ± SEM, n=6 (2 replicate egg from 3 fish). Significance was determined by one-way ANOVA with Bonferroni multiple comparison post-test. Different letters indicate significant differences between treatment groups in the post hoc test ($P < 0.05$).

Supplementary Figure 6

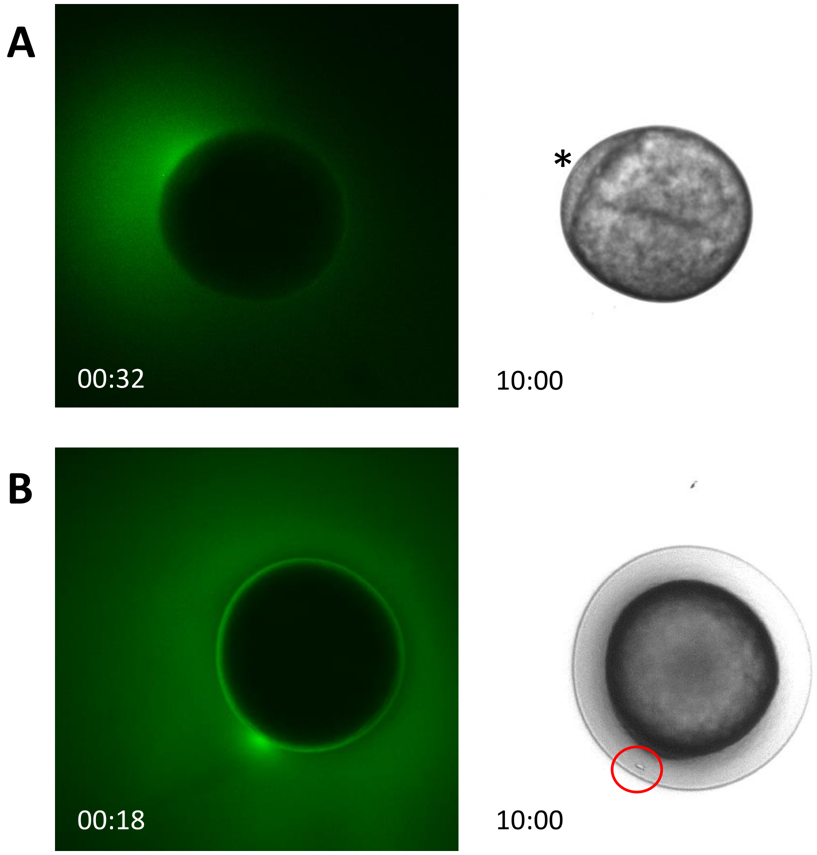

Supplementary Figure 6. Zinc exocytosis begins at the animal pole. A-B, FluoZin-3 fluorescence from WT eggs during zinc exocytosis (left) and brightfield images (right) 10 min post activation showing the blastodisc (*) and micropyle (red circle) respectively.

Description of supplemental movies

Supplemental Movie 1. Loss of zinc containing cortical vesicles upon activation of a representative wildtype egg. Scale bar: 100 µm. Images taken every 20 s for a total of 10 min.

Supplemental Movie 2. Loss of zinc containing cortical vesicles upon activation of a representative *zip9*$^{-/-}$ egg. Scale bar: 100 µm. Images taken every 20 s for a total of 10 min.

Supplemental Movie 3. Increase in extracellular zinc upon activation of a representative wildtype egg. Scale bar: 200 µm. Images taken every 2 s for a total of 10 min.

Supplemental Movie 4. Increase in extracellular zinc upon activation of a representative *zip9*$^{-/-}$ egg. Scale bar: 200 µm. Images taken every 2 s for a total of 10 min.

Supplemental Movie 5. A rise in intracellular calcium proceeds zinc exocytosis in a representative WT egg. Images taken every ~3.4 seconds for 5 min. green, extracellular zinc; purple, intracellular calcium.
